# Time budgets of dairy cows in a cow-calf contact system with automatic milking

**DOI:** 10.3168/jdsc.2023-0401

**Published:** 2023-10-16

**Authors:** Teresa Johansson, Sigrid Agenäs, Mikaela Lindberg

**Affiliations:** 1 Department of Animal Breeding and Genetics, Swedish University of Agricultural Sciences, 750 07 Uppsala, Sweden; 2 Department of Animal Nutrition and Management, Swedish University of Agricultural Sciences, 750 07 Uppsala, Sweden; 3 The Beijer Laboratory for Animal Science, Swedish University of Agricultural Sciences, Uppsala, Sweden

## Abstract

Daily time budgets can be used to determine the amount of time cows allocate to different behaviors throughout the day and can be useful when evaluating housing and management systems. There is a growing interest in keeping dairy cows and calves together during the first months of lactation; however, it is not known how their time budgets are affected by the calf contact. The aim of this study was to investigate differences in time budgets between 2 groups of dairy cows housed in the same pen within a freestall system with automatic milking. One group of cows had access to their calves until 4 mo of lactation, whereas the other group had no direct contact with the calves. Using focal animal sampling by video analysis and sensor data available from the milking unit we determined the 24-h time budgets of 37 dairy cows. The sample consisted of 20 primi- and 17 multiparous cows of the breeds Swedish Red and Swedish Holstein. The cows were randomly assigned either cow-calf contact or no contact, with separation from the calf within 24 h postparturition. Data were collected when cows were on average 43, 75, and 107 d in milk. The results showed that the cow-calf cows spent less time eating silage, without reduced dry matter intake, less time socializing with other cows, and less time standing in cubicles. However, the duration spent in the waiting area in front of the milking unit was greater among the cow-calf cows. In conclusion, neither cow-calf contact nor no-contact cows seemed to be constrained in any of the included behaviors to a point of reduced welfare; hence, a cow-calf contact system in combination with automatic milking may be possible to set up on farms with maintained or improved animal welfare.

## Highlights

• Time budgets of 37 dairy cows were determined: 19 with calf contact until 4 months of lactation, and 18 separated from the calf within 24 hours of parturition.
• Dairy cows with calf contact spent less time eating silage without reduced dry matter intake.
• Dairy cows without calf contact spent more time standing in cubicles.
• Dairy cows with calf contact spent more time waiting to be milked.

## Graphical Abstract

**Figure:**
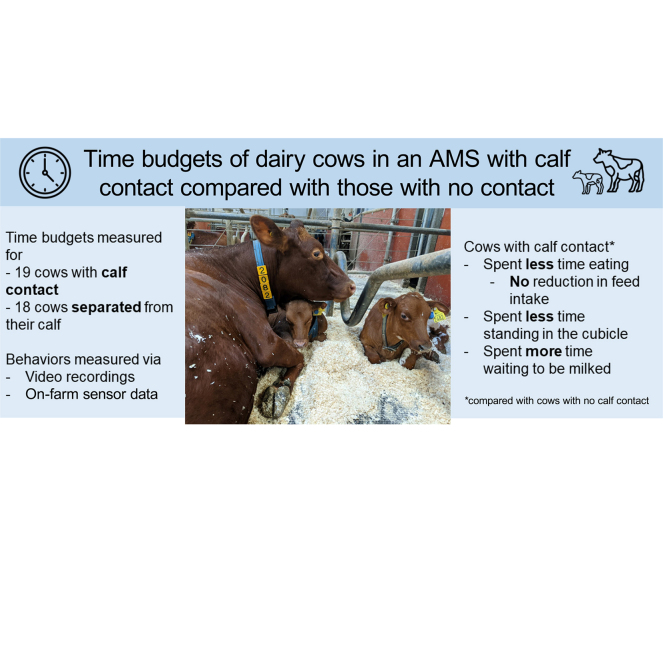
**Summary:** Time budgets were assessed in 20 primi- and 17 multiparous cows in a cow-calf study until 4 months of lactation. The cows were housed in the same automatic milking system (AMS) pen, and randomly assigned either to stay with the calf or to separate within 24 hours postparturition. The results of the study showed that certain behaviors were reduced in duration for the cow-calf group: time eating silage, socializing with other cows, and standing in cubicles. The cow-calf cows also spent more time in the waiting area in front of the milking unit. However, neither of the treatments seemed to constrain any activities to the point of reduced welfare for the animals. In conclusion, the addition of calves to a loose housing system with automatic milking is possible.

In modern dairy farming cows and calves are separated within the first 24 h after the calf is born. However, interest in dairy farming with cow and calf contact (**CCC**) that allows suckling during the milk feeding period is growing among the public, dairy farmers, and industry (Agenäs, 2017; Beaver et al., 2019; Meagher et al., 2019). Arguments used to justify immediate separation of the dam and the calf include control over colostrum consumption (Franklin et al., 2003), reduction of disease transmission (Windsor and Whittington, 2010), and decreased distress at early separation compared with separation at d 4, 7, and 14 (Flower and Weary, 2001; Stěhulová et al., 2008).

Time budgets of dairy cows in loose housing systems can be defined as the allocation of behaviors over a 24-h period, such as lying, feeding, socializing, drinking, and milking. The time budget of dairy cows provides valuable insight into whether the cow is performing essential activities, such as lying and eating, for adequate amounts daily as these behaviors are crucial to welfare and production of the dairy cow (Helmreich et al., 2014). The cow has little control over the time spent in milking facilities as this is largely decided by the management and milking system. However, an automatic milking system (**AMS**) is supposed to offer the cows a high degree of control of how other activities are distributed over the day. Time is a limited resource for the dairy cow in a loose housing system (Løvendahl and Munksgaard 2016) with different factors, such as queuing to feed stations and automatic milking units, causing time constraints (Helmreich et al., 2014) in daily behaviors. External factors (e.g., housing and management; Gomez and Cooke, 2010), as well as internal factors (e.g., lactation and milk yield; Løvendahl and Munksgaard, 2016), affect the time budgets.

Although it has been shown that CCC allows more natural behaviors (Johnsen et al., 2021) and that calves show less stereotypies in these systems (Fröberg and Lidfors, 2009), effects of CCC systems on cow time budgets have not been studied. As dairy cows will perform maternal behaviors when given the opportunity to do so (Jensen, 2011), there is a risk that the demands for time spent on eating, rumination, and resting in high-yielding dairy cows may be compromised if time is also allocated toward interactions with the calf. Allocation of behaviors throughout the day may need to be adjusted to perform maternal behaviors, such as licking, nursing, and staying close to the calf (Wenker et al., 2020), in a CCC system.

The aim of this study was to investigate differences in time budgets between dairy cows housed with calf contact (CCC) or no calf contact (**NC**) in the same automatic milking unit pen. We hypothesized that the overall time budgets between CCC and NC cows would differ. We expected CCC cows to use part of their time budget to interact with their calf and perform maternal behaviors, thus requiring adjustments to their overall time budget, reducing time spent on other behaviors.

The study was carried out at the Swedish University of Agricultural Sciences dairy herd for education and research (Lövsta Lantbruksforskning, Uppsala, Sweden). All procedures involving animals were approved by Uppsala local ethics committee (ID no. 5.818–18138/2019). Swedish animal welfare legislation was followed throughout the housing and care of the animals.

The herd averages just under one calving per day. Cows and their newborn calves were enrolled in the trial during a 6-wk period, between September 1 and October 15, 2020. When a calf was born, the cow and calf pair was either assigned to one of the 2 treatment groups (NC and CCC); every other calf of each sex was kept with the dam and the rest of the calves were separated on the day of birth while balancing the groups for calf, dam breed, and dam parity. Thus, pseudorandomization was used to achieve balanced groups, due to the limited number of animals. Forty cow-calf pairs were recruited to the study. However, 2 of the recruited CCC pairs and 1 NC pair were removed before the start of collection of data for time budgets due to health problems unrelated to the study. Therefore, the study included 13 Swedish Holstein (**SH**) and 24 Swedish Red breed (**SRB**) dairy cows, distributed as 9 NC (4 SH, 5 SRB) and 11 CCC (4 SH, 7 SRB) primiparous dams, and 9 NC (2 SH, 7 SRB) and 8 CCC (3 SH, 5 SRB) multiparous dams. The sexes of the calves in the CCC group were 7 males and 12 females. Both cows and calves were fitted with ear tags that were read at the selection gates, feeding tables, concentrate feeders, and the milking unit (**MU**). All cows were also equipped with activity sensors, on the right hind leg, for collection of data on individual lying time, number of lying bouts, and length of lying bouts.

A few days prepartum, cows were moved to and housed in individual 12.6 m^2^ calving boxes with a rubber mat and wood shavings as bedding. Following the first milking the cows were milked twice daily, at approximately 0500 and 1600 h. The NC calves were separated from their dams within 24 h postpartum and housed in individual calf pens (1.1 m^2^ with straw bedding) for 3 to 4 d before moving into group pens (23.4 ± 7.34 m^2^) with wood shavings as bedding and 4 to 5 calves per pen in the calf barn. Their dams stayed in the calving box for 3 d before moving to the AMS. The CCC pairs remained in the calving boxes for 3 d, after which they were moved to the AMS.

All experimental animals were housed in the same pen within a freestall system in an insulated and ventilated barn with “feed first” cow traffic system and AMS. The total number of cows in the pen was 57 to 58 during the trial, including cows that were not part of the study. The cows were able to move freely from the cubicle areas to the silage area (Figure 1). In the silage area (A) cows shared 20 silage bins, 7 water cups, and 2 cow comfort brushes. The cow traffic was semi-guided with the use of a 3-way selection gate from the silage area (A) to the waiting area in front of the MU, to cubicles (D/E) or to the contact area (C) if they belonged to treatment CCC. Calves did not have access to (D/E) and the silage area (A); thus, the NC cows did not have any physical contact with the calves. Calves had access to a 73 m^2^ calf creep (B) placed outside the pen adjacent to the cubicles (C/D). Between the selection gate (a) and the full contact cubicle area, CCC cows walked through a spring-loaded gate (b). The same type of gate (c) was placed at the exit from the full-contact cubicle area to no-contact cubicles. From that area all cows could move freely to the partial contact cubicle area (D) or pass a one-way gate (d) to the silage area. Details on the experimental facility and management have been reported by Wegner and Ternman (2023). When guided to the waiting area before the MU, the cows voluntarily enter the MU, one at a time, to be milked. Once milking was completed, the cow exits the MU back into the feeding area. The daily mean milk yield for the NC cows was 37.8 kg and for the CCC cows the mean was 21.2 kg (*P* < 0.001; SEM = 1.41).

**Figure 1:**
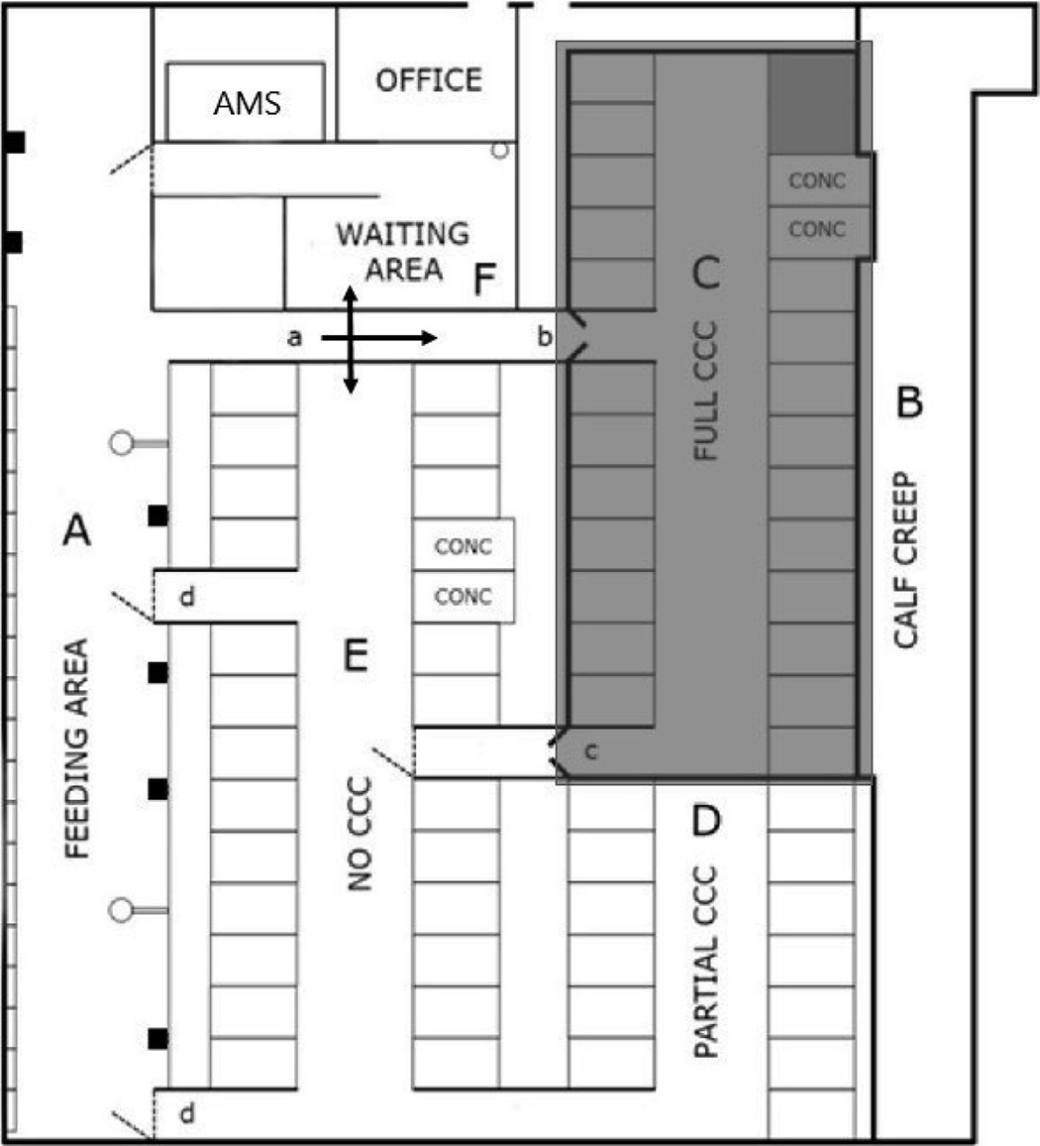
Drawing of the automatic milking system (AMS) barn. (A) Roughage area, (B) calf creep area, (C) full cow-calf contact (CCC) cubicle area, (D) partial CCC cubicle area, (E) no CCC cubicle area, and (F) AMS waiting area. Gates: (a) selection gate, (b and c) spring-loaded gates, and (d) one-way gates. The gray area indicates the area only CCC can access.

The cows had ad libitum access to grass-clover silage fed in troughs equipped with scales allowing for registration of each individual's feed intake. The concentrate was fed in 4 automatic feeding stations and allocated according to expected individual milk yield based on herd data. It was not downregulated for CCC dams even if milk yield to the MU was lower than the expected yield due to the milk consumed by the calf. Of the total daily concentrate intake, 1 to 3 kg of concentrate was distributed during 24 h in the milking robot depending on number of visits.

Eight cameras were positioned above the AMS and video footage was recorded continuously in the experimental areas. Cow activities were observed from the videos at 3 time points of the study, 5 wk apart (November 5–7, 2020; December 10–12, 2020; and January 14–16, 2021). At each time point video material from 48 h was used. The cows were marked with unique symbols with animal-safe paint to distinguish individuals during video analysis. The video analysis started at 1900 h the day the cows were marked. Time budgets of the cows were determined by performing animal sampling of the behavior of each cow in the experiment every 10 min (Mitlöhner et al., 2001) for the 48-h period. The behaviors were defined and recorded using the ethogram (Table 1).

**Table 1:** Definition of behaviors; ethogram used to record behaviors for the time budget

Behavior	Definition
Eating silage	The cow's head is in the feed trough containing silage and the cow's muzzle is touching the silage.
Eating concentrates	The cow's head is over the automatic concentrate feeder.
Standing in the waiting area	The cow has passed the selection gate and is standing in the automatic milking system waiting area.
Standing by the brush	The cow is standing next to the brush and some part of the body is being touched by the brush.
Standing in the cubicle	The cow is standing with 2, 3, or 4 legs in the stall.
Standing or walking in the feed aisles	The cow is standing idle (i.e., not moving or interacting physically) or walking (i.e., moving one or more legs) in the silage feed area.
Standing or walking in the cubicle aisles	Standing idle (i.e., not moving or interacting physically) or walking (i.e., moving one or more legs) in the cubicle area.
Social interactions with another cow	Any form of physical contact with another cow (i.e., any part of the cow in contact with any part of another cow).
Social interactions with a calf	Physical contact with the calf (i.e., any part of the cow in contact with any part of the calf).
Suckled by a calf	The calf is underneath the back half of the cow near the udders.
Lying in a stall	The cow's body in contact with the floor in the stall.
Drinking	The cow's head is over the water bowl.
Milking	The cow is in the milking robot.

The registration of selection gate passages is continuously recorded for all animals. We collected data from the selection gate that directs the individuals into the MU waiting area, and data from when they enter the MU to be milked to determine the amount of time spent in the waiting area for each cow. Data were also collected from the automatic recordings of milk yield and feed intake continuously, then divided into three 5-wk periods corresponding to the time of analyses of time budgets (October 4 to November 7, 2020; November 8 to December 12, 2020; and December 13, 2020, to January 16, 2021).

The data were analyzed using SAS 9.4 (SAS Institute Inc., Cary, NC). The time budgets were summarized over the 48 h of observation, divided by 2 before the statistical analyses to get each cow's time budget calculated as the duration of each behavior in minutes per 24-h period. This was to ensure that animal behaviors that do not occur in 24-h cycles were captured. In all analyses, primiparous cows formed one lactation class and multiparous cows formed one lactation class. All fixed effects were kept in the final model even if they were not significant, and all the possible interactions between the variables were tested and then removed if shown to not be significant. Effects were considered to be significant if *P* < 0.05. Data of time budgets were subjected to the Glimmix procedure with a Poisson distribution in SAS using the following model:

[1] Y_ijklm_ = µ + C_i_ + P_j_ + B_k_ + L_l_ + T_m_ + P_j_B_k_ + P_j_L_l_ + P_j_T_m_ + B_k_L_l_ + B_k_T_m_ + L_l_T_m_ + P_j_B_k_L_l_ + P_j_B_k_T_m_ + B_k_L_l_T_m_ + P_j_B_k_L_l_T_m_ + ε_ijklm_,

where Y_ijklm_ is the dependent variable, µ is the overall mean, C_i_ is the random effect of cow, P_j_ is the fixed effect of observation period, B_k_ is the fixed effect of breed, L_l_ is the fixed effect of lactation class, T_m_ is the fixed effect of treatment, and ε_ijklm_ is the random error. For the behaviors socializing with a calf or suckled by a calf, the differences between treatments could not be analyzed as only the CCC cows had the possibility to perform these behaviors, and they were therefore analyzed using the same model [1] but without the treatment variable.

Data collected from the activity sensors were log_10_ transformed due to the data set being skewed, and then transformed back using antilog_10_. Data collected from the activity sensors, time in waiting area, milk yield, and feed intake were inserted into the data set as means per cow and day. Analysis of variance was then performed using the Mixed procedure in SAS, using model [1].

Time budgets for the NC and CCC cows are presented in Table 2. No significant interactions were found. Cows without calf contact spent more time eating silage than the CCC cows, but the amount in kilograms of DMI did not differ between the treatments. The shorter eating time observed in the time budget analyses with the same DMI in CCC cows is in line with other work showing that the DMI can be maintained when time constraints are placed on dairy cows (Munksgaard et al., 2005). In the study by Munksgaard et al. (2005), cows increased their feed intake rate to compensate for the reduced amount of time that they had to feed throughout the day regardless of lactation stage (40–60 DIM or 257–276 DIM). The time spent eating increased in both treatments as the study progressed, similar to previous studies; in a full lactation study, Nielsen et al. (2000) saw an increase in the eating time as the lactation period continued. According to Dijkstra et al. (2012) dairy cows that are housed indoors typically spend 4 to 7 h eating per day, which is longer than both treatment groups spent. There are several factors that could play a role in this, perhaps the type of feed provided (Nielsen et al., 2000), the frequency of feed delivery, the type of traffic used, or the priorities of the animals (von Keyserlingk and Weary, 2010).

**Table 2:** Time budget results for dairy cows without (NC) and with (CCC) calves present in the automatic milking system (AMS; minutes per 24 h; % of total time within parentheses)

Behavior	Treatment		SEM^2^	*P*-value^1^			
	NC	CCC		Treatment	Lactation	Breed	Period
Eating silage	155 (11.0)	136 (9.7)	6.5	0.025	0.87	0.29	<0.001
Eating concentrates	59 (4.2)	53 (3.8)	2.5	0.069	0.53	0.13	0.070
AMS waiting area	68 (4.8)	157 (11.2)	17.0	<0.001	0.52	0.53	0.002
Standing by the brush	19 (1.3)	21 (1.5)	3.1	0.49	0.15	0.44	0.23
Standing in a cubicle	158 (11.2)	126 (9.0)	11.3	0.027	0.45	0.025	0.001
Standing or walking in feed aisles	106 (7.5)	93 (6.6)	13.3	0.46	0.11	0.36	0.011
Standing or walking in cubicle aisles	58 (4.1)	56 (4.0)	4.6	0.73	0.41	0.039	0.023
Socializing with cow	3.3 (0.2)	1.1 (0.1)	0.92	0.007	0.46	0.53	<0.001
Socializing with calf	0.0	13^3^ (0.9)	—	—	0.51	0.28	0.92
Suckled by a calf	0.0	17^3^ (1.2)	—	—	0.90	0.31	0.089
Lying in a stall	742 (52.5)	690 (49.2)	21.7	0.073	0.49	0.20	0.0012
Drinking	22 (1.6)	17 (1.2)	2.7	0.19	0.29	0.57	0.05
In AMS, milking	22 (1.6)	23 (1.6)	1.2	0.61	0.92	0.99	0.52

1 Probability of significant effect of treatment (CCC/NC), lactation class (first or older), breed (Swedish Holstein or Swedish Red), and period (1–3). No significant interactions were found (*P* > 0.05).

2 SEM = standard error of the means for treatment.

3 Mean values.

The amount of time spent waiting to be milked in front of the MU was verified using data from the selection gates. These data showed a difference between the treatments (*P* < 0.001; SEM = 0.22). The CCC cows spent a mean of 2.4 h/d in the waiting area compared with the NC cows spending a mean of 1.2 h/d waiting. The CCC cows visited the MU 2.4 times per day, whereas the NC cows visited 2.2 times (*P* = 0.049; SEM = 0.06). The amount of time cows in both treatments spent in the waiting area is comparable to previous studies. When comparing free and forced traffic, Munksgaard et al. (2011) observed the cows waiting an average 1.5 h/d to enter the MU; furthermore, there was a large individual variation seen in that study which was also observed in the current study. The variation between individuals might be explained by the hierarchy of the herd. Meijering et al. (2004) and Halachmi (2009) observed lower ranked cows waiting longer durations in the waiting area compared with cows of higher ranks. However, the ranks of the individuals in our study were not determined. According to Halachmi (2009), primiparous cows are usually of low rank; therefore, the similar distribution of primiparous and multiparous cows in our study may have mitigated this effect, since no effects of parity were shown in our analyses of data in the waiting area. Due to the current setup of the pen, the position of the MU in relation to the contact area may explain at least part of the longer waiting times for the CCC cows. The contact area and calf creep were positioned behind the waiting area, meaning that if in queue for milking the cows would be facing away from the calves. Some individuals in the CCC treatment occasionally stood at the edge of the waiting area looking toward the calves as opposed to getting in the queue for the MU, leading to waiting times that were longer than necessary. The cows' calf-directed behavior seems to be positively correlated with the amount of time the cow-calf pair spend together (Wenker et al., 2020, 2021; Johnsen et al., 2021).

There was a tendency (*P* = 0.07) for CCC cows to spend less time lying compared with the NC cows, and both treatments spent more time lying during the third observation period. The data recorded from the activity sensors showed that the NC cows had an average lying time of 12.3 h/d and the CCC cows had an average lying time of 11.6 h/d (*P* = 0.18; SEM = 0.80). This was accomplished with an average of 12.8 lying bouts for both treatments (*P* = 0.99; SEM = 1.08). Lying times observed for both treatments were comparable to those reported in other studies, Jensen et al. (2005) and Munksgaard et al. (2005), where they suggest that approximately 12 h of lying time throughout each 24-h period is optimal for dairy cows. Other studies have also shown lying times increasing as the lactation period continues (Bewley et al., 2010), likely due to the changes in the milk yield that occur throughout the lactation (Norring et al., 2012).

In conclusion, there were differences seen in the time budgets of dairy cows with their calf present in the loose housing system with an AMS compared with the cows that were separated from their calves following parturition. Cows with their calf present spent less time eating silage, socializing with other cows, and standing in the cubicles compared with cows without calves. Instead, the cows with calves spent more time in the waiting area queuing to be milked. However, the extent to which time budgets of NC cows were affected by having calves nearby cannot be determined from this study. The time spent engaged in each of the recorded behaviors was the same, with a few minor exceptions, when cows were housed in a freestall system with and without their calf; reported means were also similar to values presented in previous studies. Under the conditions described in the present study, it appears that keeping cows and calves together may be possible from the perspective of the cow's time budget; however, many questions regarding the myriad of other aspects associated with a cow-calf contact system, such as duration of contact and how best to wean, must also be addressed.

## References

[bib1] Agenäs S. (2017). Editorial: We need to bring the calves back to the dairy cows. J. Dairy Res..

[bib2] Beaver A., Meagher R.K., von Keyserlingk M.A.G., Weary D.M. (2019). Invited review: A systematic review of the effects of early separation on dairy cow and calf health. J. Dairy Sci..

[bib3] Bewley J.M., Boyce R.E., Hockin J., Munksgaard L., Eicher S.D., Einstein M.E., Schutz M.M. (2010). Influence of milk yield, stage of lactation, and body condition on dairy cattle lying behaviour measured using an automated activity monitoring sensor. J. Dairy Res..

[bib4] Dijkstra C., Veermäe I., Praks J., Poikalainen V., Arney D.R. (2012). Dairy cow behavior and welfare implications of time waiting before entry into the milking parlor. J. Appl. Anim. Welf. Sci..

[bib5] Flower F.C., Weary D.M. (2001). Effects of early separation on the dairy cow and calf: 2. Separation at 1 day and 2 weeks after birth. Appl. Anim. Behav. Sci..

[bib6] Franklin S.T., Amaral-Phillips D.M., Jackson J.A., Campbell A.A. (2003). Health and performance of Holstein calves that suckled or were hand-fed colostrum and were fed one of three physical forms of starter. J. Dairy Sci..

[bib7] Fröberg S., Lidfors L. (2009). Behaviour of dairy calves suckling the dam in a barn with automatic milking or being fed milk substitute from an automatic feeder in a group pen. Appl. Anim. Behav. Sci..

[bib8] Gomez A., Cook N.B. (2010). Time budgets of lactating dairy cattle in commercial freestall herds. J. Dairy Sci..

[bib9] Halachmi I. (2009). Simulating the hierarchical order and cow queue length in an automatic milking system. Biosyst. Eng..

[bib10] Helmreich S., Hauser R., Jungbluth T., Wechsler B., Gygax L. (2014). Time-budget constraints for cows with high milking frequency on farms with automatic milking systems. Livest. Sci..

[bib11] Jensen M.B. (2011). The early behaviour of cow and calf in an individual calving pen. Appl. Anim. Behav. Sci..

[bib12] Jensen M.B., Pedersen L.J., Munksgaard L. (2005). The effect of reward duration on demand functions for rest in dairy heifers and lying requirements as measured by demand functions. Appl. Anim. Behav. Sci..

[bib13] Johnsen J.F., Johanssen J., Aaby A., Kischel S., Ruud L., Soki-Makilutila A., Bjørklund Kristiansen T., Gladsø Wibe A., Bøe K.-E., Ferneborg S. (2021). Investigating cow−calf contact in cow-driven systems: Behaviour of the dairy cow and calf. J. Dairy Res..

[bib14] Løvendahl P., Munksgaard L. (2016). An investigation into genetic and phenotypic variation in time budgets and yield of dairy cows. J. Dairy Sci..

[bib15] Meagher R.K., Beaver A., Weary D.M., von Keyserlingk M.A.G. (2019). Invited review: A systematic review of the effects of prolonged cow–calf contact on behavior, welfare, and productivity. J. Dairy Sci..

[bib16] Meijering A., Hogeveen H., de Koning C.J.A.M. (2004). Automatic milking, a better understanding.

[bib17] Mitlöhner F.M., Morrow-Tesch J.L., Wilson S.C., Dailey J.W., McGlone J.J. (2001). Behavioral sampling techniques for feedlot cattle. J. Anim. Sci..

[bib18] Munksgaard L., Jensen M.B., Pedersen L.J., Hansen S.W., Matthews L. (2005). Quantifying behavioural priorities—effects of time constraints on behaviour of dairy cows, *Bos taurus*. Appl. Anim. Behav. Sci..

[bib19] Munksgaard L., Rushen J., de Passillé A.M., Krohn C.C. (2011). Forced versus free traffic in an automated milking system. Livest. Sci..

[bib20] Nielsen B.L., Veerkamp R.F., Lawrence A.B. (2000). Effects of genotype, feed type and lactational stage on the time budget of dairy cows. Acta Agric. Scand. A Anim. Sci..

[bib21] Norring M., Valros A., Munksgaard L. (2012). Milk yield affects time budget of dairy cows in tie-stalls. J. Dairy Sci..

[bib22] Stěhulová I., Lidfors L., Špinka M. (2008). Response of dairy cows and calves to early separation: Effect of calf age and visual and auditory contact after separation. Appl. Anim. Behav. Sci..

[bib23] von Keyserlingk M.A.G., Weary D.M. (2010). Review: Feeding behaviour of dairy cattle: Measures and applications. Can. J. Anim. Sci..

[bib24] Wegner C.S., Ternman E. (2023). Lying behaviour of lactating dairy cows in a cow-calf contact freestall system. Appl. Anim. Behav. Sci..

[bib25] Wenker M.L., Bokkers E.A., Lecorps B., von Keyserlingk M.A., van Reenen C.G., Verwer C.M., Weary D.M. (2020). Effect of cow-calf contact on cow motivation to reunite with their calf. Sci. Rep..

[bib26] Wenker M.L., van Reenen C.G., de Oliveira D., McCrea K., Verwer C.M., Bokkers E.A. (2021). Calf-directed affiliative behaviour of dairy cows in two types of cow-calf contact systems. Appl. Anim. Behav. Sci..

[bib27] Windsor P.A., Whittington R.J. (2010). Evidence for age susceptibility of cattle to Johne's disease. Vet. J..

